# Oral minoxidil 7.5 mg for hair loss increases heart rate with no change in blood pressure in 24 h Holter and 24 h ambulatory blood pressure monitoring^☆^

**DOI:** 10.1016/j.abd.2023.08.016

**Published:** 2024-05-20

**Authors:** Baltazar Dias Sanabria, Yuri Chiarelli Perdomo, Hélio Amante Miot, Paulo Müller Ramos

**Affiliations:** a Dermatology Outpatient Clinic, Universidade Federal do Mato Grosso do Sul, Campo Grande, MS, Brazil; b Department of Dermatologics, Infectious and Parasitic Diseases, Faculdade de Medicina de São José do Rio Preto, São José do Rio Preto, SP, Brazil; c Department of Infectology, Dermatology, Imaging Diagnosis and Radiotherapy, Universidade Estadual Paulista, Botucatu, SP, Brazil

Dear Editor,

Low-dose oral minoxidil (LDOM) has emerged as an important alternative for treating different causes of hair loss.^1^ Nonetheless, its cardiovascular adverse effects, such as tachycardia, hypotension, and edema, remain a concern even at low doses.

The standard dose for the treatment of hypertension typically ranges from 10 to 40 mg/day, and there is no consensus about the ideal dosage for treating hair loss.^2^

A wide range of doses (from 0.25 to 5 mg/day) has been evaluated in clinical studies, but not exceeding 5 mg/day.^2^ Recently, a metanalysis demonstrated a positive dose-dependent association of LDOM with an increase in hair density as well as adverse effects.^2^

We have recently assessed 30 adult males taking 5 mg oral minoxidil for androgenetic alopecia (AGA) with 24-h Holter monitoring and 24-h ambulatory blood pressure monitoring (ABPM) before and after 24 weeks of treatment. They presented no relevant alterations regarding 24-h Holter monitoring and ABPM.^3^ These findings were reinforced by an evaluation of 10 men with ABPM at baseline and after the first dose of 5 mg oral minoxidil.^4^

Previous pharmacokinetics studies have shown a mild reduction in blood pressure and a slight increase in heart rate in normotensive patients using oral minoxidil at doses up to 10 mg/day.^5^ To assess the potential cardiovascular adverse effects of higher doses of oral minoxidil for hair loss, we increased the dose from 5 to 7.5 mg/day in 11 of the 30 patients who had completed our prior study. After 6-weeks of taking the increased dose, we re-evaluated these patients using 24-h Holter monitoring and ABPM.

The main clinical and demographic data of the participants are presented in Table 1. The ABPM and Holter monitoring results are displayed in Table 2. Despite a subclinical increase in the heart rate, oral 7.5 mg/day minoxidil did not lead to hypotension, tachycardia, or impairment in the nighttime dip.

**Table 1:** Main clinical and demographic data from the 11 participants of the study.

Variables		Values
Age (years), mean (SD)		37.9 (7.7)

Weight (kg), mean (SD)		86.2 (13.7)

Ethnicity, n (%)	White	8 (73%)
	Brown	2 (18%)
	Black	1 (9%)

Concomitant use drugs, n (%)	Finasteride	2 (18%)

**Table 2:** Main results of 24 h Holter monitoring and 24 h ambulatory blood pressure monitoring of 11 adult males with androgenetic alopecia assessed before (T0), after 24 weeks (T24) of treatment with 5 mg/d oral minoxidil, and after 6-weeks (T30) of treatment with 7.5 mg/d oral minoxidil.

Variables	T0 (Pre-treatment)	T24 (5 mg/d)	T30 (7.5 mg/d)	p-value* (T0 × T30)	p-value* (T24 × T30)
Heart rate (24 h); bpm^a^					
Minimum	47.3 (7.4)	48.5 (6.4)	53.0 (5.5)	**0.009**	**0.024**
Average	72.6 (8.7)	75.7 (9.1)	81.3 (7.8)	**0.037**	0.067
Maximum	129.9 (15.8)	124.5 (11.1)	131.9 (17.5)	0.400	0.142
Extra-systoles (events per 24 h)^b^					
Supraventricular	3 (2–14)	4 (2–11)	2 (1–6)	0.300	0.302
Ventricular	0 (0–0)	0 (0–1)	0 (0–0)	0.416	0.330
Mean arterial pressure#; mmHg					
Daylong	90.4 (10.4)	87.0 (7.7)	89.9 (9.7)	0422	0.298
Awake	94.6 (11.2)	89.4 (8.6)	93.8 (9.8)	0.396	0.202
Sleep	77.8 (9.3)	76.9 (7.7)	80.0 (8.6)	0.337	0.338
Nighttime dip (%)	-15.6 (8.1)	-11.0 (6.3)	-14.9 (5.7)	0.372	0.136
≥ 10%	8 (73%)	3 (30%)	5 (50%)	0.096	0.016
Systolic blood pressure; mmHg^a^					
Daylong	124.1 (11.7)	121.4 (9.3)	124.9 (12.4)	0.416	0.377
Awake	127.9 (13.0)	123.5 (10.2)	128.9 (12.7)	0.368	0.268
Sleep	112.4 (11.1)	112.5 (9.3)	114.0 (10.8)	0.418	0.419
Diastolic blood pressure; mmHg^a^					
Daylong	73.6 (9.9)	69.8 (7.9)	72.4 (9.0)	0.335	0.301
Awake	77.9 (10.9)	72.4 (8.8)	76.3 (9.2)	0.291	0.202
Sleep	60.5 (8.9)	59.1 (8.4)	63.0 (8.7)	0.304	0.257

AST, Aspartate Aminotransferase; ALT, Alanine Aminotransferase; LDL, Low-Density Lipoprotein; HDL, High-Density Lipoprotein.

a Mean (SD).

b Median (p25–p75).

# (2*diastolic pressure + systolic Pressure)/3.

* One-tailed p-value from generalized mixed model, corrected (post-hoc) by Šidák procedure.

One participant referred to headache and nine hypertrichoses with oral minoxidil 5 mg/day which did not lead to treatment discontinuation. None of them presented any adverse effects like headache, tachycardia, dizziness, edema, or insomnia after increasing the dose to 7.5 mg/day.

These results reinforce the mild antihypertensive effects of oral minoxidil in normotensive individuals. However, we suggest that doses above 5 mg should not be considered the standard for hair loss treatment and should only be used in exceptional circumstances. In such cases, we recommend that clinicians increase the dose gradually rather than starting with higher doses. It is essential to consider that even very low doses (0.25 mg/day) of oral minoxidil have been associated with uncommon idiosyncratic but severe adverse effects, such as pericardial and pleural effusions.^6^

This study provides additional follow-up data from a previous cohort. Although the sample size was modest, it did not hinder the detection of major cardiovascular trends among patients taking oral minoxidil 7.5 mg/day.

In conclusion, administration of minoxidil 7.5 mg/day for AGA in normotensive adults was well tolerated and resulted in a mild increase in heart rate, with no observed changes in blood pressure.

## Financial support

None declared.

## Authors’ contributions

Baltazar Dias Sanabria: Data collection, approval of the final version of the manuscript; manuscript planning; drafting and editing of the manuscript; critical review of the literature and critical review of the manuscript.

Yuri Chiarelli Perdomo: Data collection, approval of the final version of the manuscript; manuscript planning; drafting and editing of the manuscript; critical review of the literature and critical review of the manuscript.

Hélio Amante Miot: Approval of the final version of the manuscript; manuscript planning; drafting and editing of the manuscript; critical review of the literature and critical review of the manuscript.

Paulo Müller Ramos: Approval of the final version of the manuscript; manuscript planning; drafting and editing of the manuscript; critical review of the literature and critical review of the manuscript.

## Conflicts of interest

None declared.
